# The incidence and distribution of *Listeria monocytogenes* in ready‐to‐eat vegetables in South‐Western Nigeria

**DOI:** 10.1002/fsn3.263

**Published:** 2015-07-20

**Authors:** Titilayo A. Ajayeoba, Olusegun O. Atanda, Adewale O. Obadina, Mobolaji O. Bankole, Olawale O. Adelowo

**Affiliations:** ^1^ Department of Food Science and TechnologyCollege of Food and Human EcologyFederal University of AgricultureAbeokutaOgun‐StateNigeria; ^2^ Department of Biological SciencesCollege of Natural and Applied SciencesMcPherson UniversityKm 96, Lagos‐ Ibadan ExpresswaySeriki‐SotayoAbeokutaOgun StateNigeria; ^3^ Department of MicrobiologyCollege of BiosciencesFederal University of AgricultureAbeokutaOgun‐StateNigeria; ^4^ Environmental Microbiology and Biotechnology LaboratoryDepartment of MicrobiologyUniversity of IbadanIbadanOyo StateNigeria

**Keywords:** Incidence, *Listeria monocytogenes*, ready‐to‐eat vegetables, South‐Western Nigeria

## Abstract

The study investigated the incidence of *Listeria monocytogenes* in ready‐to‐eat (RTE) vegetables: (Cucumber, *Cucumis sativas*; Cabbage, *Brassica olerecea*; Carrot, *Daucus carota*; Tomato, *Solanum lycopersicum*; Lettuce, *Lactuca sativa*) in six states in South‐Western Nigeria. A total of 555 composite samples were collected from 30 traditional markets within the states and only 244 *L. monocytogenes* species were isolated by standard methods. The incidence of *L. monocytogenes* was low and not statistically significant per vegetable type. The percentage distribution of the *L. monocytogenes* isolates in the RTE vegetables was 28.28, 9.02, 23.36, 19.67, and 19.67 for Cabbage, Carrot, Cucumber, Lettuce, and Tomatoes, respectively. Lagos state had the highest incidence of *L. monocytogenes* contamination (55%) followed by Ondo (48.89%), Oyo (48.75%), Ogun (44.09%), Osun (34.38%), and Ekiti (33.33%) states, respectively. Although the incidence of *L. monocytogenes* in the RTE vegetables in South‐Western Nigeria is low, its presence should be a source of concern as it could constitute a public health threat for its consumers.

## Introduction

Foodborne pathogens encompass a wide spectrum of microorganisms that contaminate food and water at different points during their preparation (Hanson et al. 2012). The World Health Organization (WHO) reported that 1.8 million people died in 2005 from diseases largely due to pathogens that contaminated food and water supplies (Sudershan et al. 2014). The WHO also reported that more than 200,000 people die annually of food poisoning in Nigeria (Onyeneho and Hedberg 2013). Although, the full extent of the burden and cost of unsafe food is unknown, the impact on global health, trade, and development is considered to be immense. The incidences of foodborne pathogens have been studied in Nigeria with more than 90% of annual cases of food poisoning reported to be caused by *Staphylococcus aureus, Salmonella* spp., *Clostridium perfringens, Campylobacter* spp., *Listeria monocytogenes, Vibrio parahaemolyticus, Bacillus cereus, Escherichia coli*, and *Proteus* spp. (Adetunji and Adegoke 2008; Oni et al. 2009; Enabulele et al. 2010; Eni et al. 2010; Ifeadike et al. 2012; Onyeneho and Hedberg 2013).

*Listeria monocytogenes* is a small gram‐positive, oxidase‐negative, nonsporulating motile bacterium. It is a facultative anaerobe that grows at the temperature range of −0.4 to 50°C (Bayoub et al. 2010) and at high salt concentration. The organism is capable of causing an infection known as listeriosis (Bayoub et al. 2010). *Listeria monocytogenes* is ubiquitous and had been recovered from dust, soils, water, sewage, and decaying vegetation including animal feeds and silage, from where it enters the food chain (Ikeh et al. 2010). It has also been identified to be a major pathogen that contributes to domestically acquired foodborne illnesses after nontyphodial *Salmonella* and *Toxoplasma gondii* (Centres for Disease Control, 2011). In humans, the average case‐fatality rate of listeriosis is between 20% and 30% even with adequate antimicrobial treatments (Swaminathan and Gerner‐Smidt 2007) but can be a serious invasive disease with mortality rates ranging between 80% and 99% primarily in neonates, immunocompromised adults and pregnant women causing encephalitis, septicemia, and abortion (Parihar et al. 2008).

The consumption of RTE vegetables in Nigeria has greatly increased based on their proven medical and nutritional benefits (Adeshina et al. 2012; Ieren et al. 2013). Vegetables have been identified as major vehicles for listeriosis due to their direct contamination with decaying vegetation, soil surfaces, rivers, canal waters, and effluents from sewage treatments, improper harvesting and handling procedures, improper sanitary conditions of equipment, and transportation practices (Beuchat 1996 and Donnelly 2001). Furthermore, the farm practices and handling of RTE vegetables by local farmers in Nigeria differ significantly from the Western world due to continual use of untreated waste water and animal feces as manures to fertilize plants for production of fruits and vegetables leading to microbial contamination (Amoah et al. 2009).

Even though, there are few published report on the occurrence of *L. monocytogenes* in water, food samples, and environmental sources in Nigeria (Adetunji and Adegoke 2008; Salihu et al. 2008; Ikeh et al. 2010; Nwachukwu et al. 2010), listeriosis is not a reportable disease in Nigerian health program. Furthermore, epidemiological studies, that suggest RTE foods can contribute significantly to listeriosis, are inadequate, due to the paucity of data deficiencies in knowledge about important parameters in the food chain. In addition, literature lacks information on the incidence of *L. monocytogenes* in vegetables that require no further processing (RTE).

This study therefore reports the incidence and distribution of *L. monocytogenes* in some RTE vegetables in South‐Western Nigeria.

## Materials and Methods

### Study locations

The study areas were the six states of South‐Western Nigeria. The locations lie between longitude 2° 31’ and 6° 00’ East and Latitude 6° 21’ and 8° 37’ N of Nigeria. Only traditional markets that were reputed for sale of RTE vegetables were sampled (Table 1).

**Table 1:** Sample location of ready‐to‐eat vegetables

State	Location of town and traditional market
Oyo	aOgbomosho b(Sabo), Oyo town (Sabo), Ibadan (Bodija), Saki (Oja‐Oba), Ibarapa (Maya)
Osun	Ikirun (Oke‐Afo), Osogbo (Igbona), Ede (Timi), Ejigbo, Ilesha (Adeti)
Lagos	Mushin (Mushin), Oshodi (Bolade), Mile 12 (Mandela), Oyingbo (Oyingbo), Ogba (Ogba)
Ogun	Odeda (Odeda), Ago‐Iwoye (Ago‐ garage), Sango‐Ota (Sabo), Abeokuta (Lafenwa), Ijebu‐ Ode (Ita‐osu)
Ondo	Akure (Isikan), Ikare (Oja‐ Oba), Ondo town (Mimiko), Iloro (Iloro), Owena (Owena)
Ekiti	Ifaki (Oja ‐Oba), Ado‐Ekiti (Oja‐ Bisi), Aisegba (Aisegba), Ijero (Ijero), Omuo (Kota)

a Town.

b Traditional market.

### Sample collection

Sampling was carried out bimonthly during the rain (July and September, 2012) and dry seasons (November, 2012 and January, 2013), respectively. Five different types of RTE vegetables: (Cucumber, *Cucumis sativas*; Cabbage, *Brassica olerecea*; Carrot, *Daucus carota*; Tomato, *Solanum lycopersicum*; Lettuce, *Lactuca sativa*) were sampled. Ten grams of each vegetable were purchased randomly from three different vendors in each market and mixed together to form a composite sample per vegetable per market. A total of 30 g sample was thus collected per vegetable per market and the number of samples collected per market per state depended on the availability of the RTE vegetable within the states. Samples were collected in sterile containers and stored at 4°C prior to analysis.

### Detection and enumeration of *L. monocytogenes*

The detection and enumeration of the organism were carried out by the method of the International Organization for Standards (ISO 11290‐1:1997/Amd.1:2004) as reported by Rapeanu et al. (2008). Briefly, 25 g of each vegetable was aseptically blended in a sterile Warring blender (Torrington, CT) for 5 min and pre‐enriched by diluting 10 g of each lot in 90 mL of one broth—Listeria (Oxoid (Basingstoke Hampshire, UK.)). The mixture was vortexed for 1 min by hand inversion and incubated without agitation at 30°C for 24 h. Ten microliters of the broth culture were aseptically inoculated into Brilliance Listeria Agar (Oxoid) which was incubated at 37°C for 24 h. Suspected colonies with blue or greenish coloration's were counted as *L. monocytogenes* and individual colonies further streaked on nutrient agar for confirmatory tests: Gram staining, catalase test, motility test, and Christie, Atkins, Munch‐Petersen test as described by Rapeanu et al. (2008), *β* – hemolytic activity and sugar fermentation tests as described by Dabrowski et al. (2003).

### Distribution of *L. monocytogenes* per vegetable type

The distribution of *L. monocytogenes* per vegetable type was calculated by dividing the incidence of *L. monocytogenes* per vegetable type by the cumulative number of *L. monocytogenes*‐positive isolates in the markets; which was then multiplied by 100 and expressed as the percentage distribution.

### Statistical analysis

The incidence of *L. monocytogenes* in the RTE vegetables in the markets and the states was compared statistically with SAS (Statistical Analysis Software 92.2) using Pearson chi‐square analysis. Incidences were considered statistically significant at *P* ≤ 0.05.

## Results

### Incidence and distribution of *L. monocytogenes* in South‐Western Nigeria

The incidence of *L. monocytogenes* in RTE vegetables in the traditional markets (Table 2) was generally low and was below the European Union limit of 100 cfu/g (EU, 2007). Furthermore, the incidence of *L. monocytogenes* was not significantly different (*P* ≥ 0.05) within the markets and across the states, but the incidences were higher in some markets such as Isikan (Ondo state), Bodija (Oyo state), Mushin (Lagos state), and Lafenwa (Ogun state). In contrast, *L. monocytogenes* was not isolated in some markets such as Owena (Ondo state), Ejigbo (Osun state), Oja‐Oba (Oyo state), Oja Odeda, Ago‐Garage (Ogun state), and Kota (Ekiti state).

**Table 2:** The incidence and distribution of *Listeria monocytogenes* in ready‐to‐eat (RTE) vegetables in traditional markets in South‐Western Nigeria

State	Traditional market	Incidence of *L. monocytogenes* in RTE vegetable					Cumulative incidence of *L. monocytogenes*/market sample	*P* value
		Cabbage	Carrot	Cucumber	Lettuce	Tomato		
Ondo	Isikan	4 (1.64)^a^	1 (0.41)	3 (1.23)	4 (1.64)	5 (2.05)	17/18 (94.44)	0.59
	Oja‐oba	4 (1.64)	1 (0.41)	3 (1.23)	2 (0.82)	2 (0.82)	12/20 (60.00)	
	Mimiko	0 (0.00)	1 (0.41)	2 (0.82)	3 (1.23)	2 (0.82)	8/19 (42.12)	
	Iloro	4 (1.64)	1 (0.41)	0 (0.00)	0 (0.00)	2 (0.82)	7/18 (38.89)	
	Owena	0 (0.00)	0 (0.00)	0 (0.00)	0 (0.00)	0 (0.00)	0/15 (0.00)	
X^b^		12 (4.92)	4 (1.64)	8 (3.28)	9 (3.69)	11 (3.69)	44/90 (48.89)	
Osun	Oke‐Afo	3 (1.23)	0 (0.00)	2 (0.82)	2 (0.82)	0 (0.00)	7/19 (36.84)	0.85
	Igbona	3 (1.23)	2 (0.82)	3 (1.23)	4 (1.64)	2 (0.82)	14/20 (70.00)	
	Timi	2 (0.82)	0 (0.00)	1 (0.41)	2 (0.82)	2 (0.82)	7/18 (38.89)	
	Adeti	2 (0.82)	1 (0.41)	1 (0.41)	0 (0.00)	1 (2.08)	5/19 (26.32)	
	Ejigbo	0 (0.00)	0 (0.00)	0 (0.00)	0 (0.00)	0 (0.00)	0/20 (0.00)	
X^b^		10 (4.10)	3 (1.23)	7 (2.86)	8 (3.28)	5 (2.05)	33/96 (34.38)	
Oyo	Sabo (Ogbomoso)	3 (1.23)	2 (0.82)	6 (2.46)	1 (2.08)	0 (0.00)	12/19 (63.16)	0.35
	Bodija	8 (3.28)	1 (0.41)	5 (2.05)	4 (1.64)	6 (2.46)	24/20 (120.00)	
	Sabo (Oyo Town	0 (0.00)	0 (0.00)	0 (0.00)	0 (0.00)	1 (2.08)	1/16 (6.25)	
	Oja‐Oba (Saki)	0 (0.00)	0 (0.00)	0 (0.00)	0 (0.00)	0 (0.00)	0/11 (0.00)	
	Maya	0 (0.00)	0 (0.00)	1 (0.41)	0 (0.00)	1 (0.41)	2/14 (14.26)	
X^b^		11 (4.51)	3 (1.23)	12 (4.92)	5 (2.05)	8 (3.28)	39/80 (48.75)	
Lagos	Mushin	6 (2.46)	2 (0.82)	4 (1.64)	5 (2.05)	4 (1.64)	21/20 (105.00)	0.72
	Bolade	5 (2.05)	1 (0.41)	1 (0.41)	1 (2.08)	2 (0.82)	10/20 (50.00)	
	Ogba	0 (0.00)	1 (0.41)	1 (0.41)	0 (0.00)	2 (0.82)	4/20 (20.00)	
	Oyingbo	5 (2.05)	0 (0.00)	1 (0.41)	2 (0.82)	3 (1.23)	11/20 (55.00)	
	Mandela	2 (0.82)	2 (0.82)	2 (0.82)	0 (0.00)	3 (1.23)	9/20 (45.00)	
X^b^		18 (7.38)	6 (2.46)	9 (3.69)	8 (3.28)	14 (5.74)	55/100 (55.00)	
Ogun	Oja Odeda	0 (0.00)	0 (0.00)	0 (0.00)	0 (0.00)	0 (0.00)	0/17 (0.00)	0.97
	Ago‐garage	0 (0.00)	0 (0.00)	0 (0.00)	0 (0.00)	0 (0.00)	0/19 (0.00)	
	Ita‐osu	2 (0.82)	0 (0.00)	1 (0.41)	1 (0.41)	0 (0.00)	4/20 (20.00)	
	Sabo (Sango Ota)	5 (2.05)	2 (0.82)	3 (1.23)	3 (1.23)	2 (0.82)	15/20 (75.00)	
	Lafenwa	6 (2.46)	3 (1.23)	4 (1.64)	5 (2.05)	4 (1.64)	22/20 (110.00)	
X^b^		13 (5.33)	5 (2.05)	8 (3.28)	9 (3.69)	6 (2.46)	41/93 (44.09)	
Ekiti	Oja –Oba (Ifaki)	1 (0.41)	0 (0.00)	3 (1.23)	3 (1.23)	1 (0.41)	8/20 (40.00)	0.72
	Oja Bisi	1 (0.41)	0 (0.00)	3 (1.23)	3 (1.23)	1 (0.41)	8/20 (40.00)	
	Aisegba	0 (0.00)	1 (0.41)	3 (1.23)	1 (2.08)	1 (0.41)	6/20 (30.00)	
	Kota	0 (0.00)	0 (0.00)	0 (0.00)	0 (0.00)	0 (0.00)	0/20 (0.00)	
	Otun	3 (1.23)	0 (0.00)	4 (1.64)	2 (0.82)	1 (0.41)	10/13 (76.92)	
X^b^		5 (2.05)	1 (0.41)	13 (5.33)	9 (3.69)	4 (1.64)	32/96 (33.33)	
Σ^c^		69 (28.28)	22 (9.02)	57 (23.36)	48 (19.67)	48 (19.67)	244/555	

a Percentage distribution of *L. monocytogenes* per vegetable type.

b X Incidence of *L. monocytogenes* per state.

c Σ Incidence of *L. monocytogenes* per RTE vegetable.

Lagos state recorded the highest incidence of *L. monocytogenes* contamination in Cabbages (7.38%) and Carrots (6.46%) while Ekiti state had the least incidences of 2.05 and 0.41%, respectively. In contrast, Ekiti state had the highest incidence of *L. monocytogenes* contamination of Cucumbers (5.33%) while Osun state had the least contamination (2.86%). In addition, Ondo, Ogun, and Ekiti states had the highest incidences of *L. monocytogenes* contamination of lettuce (3.69%) while Oyo state had the least contamination (2.05%). Lagos state also had the highest incidence of *L. monocytogenes* contamination of Tomatoes (5.74%) while Ekiti state had the least contamination (1.64%). Lagos state thus had the highest incidence (55%) of *L. monocytogenes* contamination followed by Ondo (48.89%), Oyo (48.75%), Ogun (44.09%), Osun (34.38%), and Ekiti (33.33%) states, respectively. Overall, out of 555 composite market samples of RTE vegetables, only 244 isolates were positive for *L. monocytogenes* (Table 2). The percentage distribution of *L. monocytogenes* isolates in the RTE vegetables was 28.28, 9.02, 23.36, 19.67, and 19.67 for Cabbage, Carrot, Cucumber, Lettuce, and Tomatoes, respectively. Furthermore, the incidence of *L. monocytogenes* (Table 3) was significantly different (*P* ≤ 0.05) in July (a rainy season) than the other months.

**Table 3:** Monthly incidence of *Listeria monocytogenes* in ready‐to‐eat (RTE) vegetables

Month of sampling	State	Incidence of *L. monocytogenes* per RTE vegetable					Cumulative incidence^b^	*P* value
		Cabbage	Carrot	Cucumber	Lettuce	Tomato		
July	Ondo	6	3	4	1	8		0.015
	Osun	6	0	2	1	1		
	Oyo	5	1	6	2	4		
	Ogun	5	2	5	2	5		
	Lagos	10	3	4	2	10		
	Ekiti	0	0	3	3	1		
X^a^		32	9	24	11	29	105	
September	Ondo	2	0	1	1	0		
	Osun	0	0	0	1	0		
	Oyo	1	0	1	1	0		
	Ogun	2	0	2	1	1		
	Lagos	2	0	3	1	3		
	Ekiti	1	0	3	0	0		
X^a^		8	0	10	5	4	27	
November	Ondo	0	0	1	3	1		
	Osun	0	2	1	2	1		
	Oyo	1	0	1	0	1		
	Ogun	1	1	0	2	0		
	Lagos	2	1	0	1	0		
	Ekiti	1	0	0	1	1		
X^a^		5	4	3	9	4	25	
January	Ondo	3	1	2	5	2		
	Osun	4	1	4	4	3		
	Oyo	4	2	4	2	3		
	Ogun	5	2	1	4	0		
	Lagos	6	1	2	2	2		
	Ekiti	3	2	6	6	1		
X^a^		25	9	19	23	11	87	

a X Incidence of *L. monocytogenes* per RTE vegetable.

b Cumulative incidence of *L. monocytogenes* per month of sampling.

## Discussion

Fruits and vegetables are important components of healthy and balanced diets; their consumption is encouraged in many countries by government health agencies for protection against a wide range of illnesses such as cancers and cardiovascular diseases (Van Duyn and Pivonka 2000). The composition of food budgets is shifting from the consumption of grains and other staple crops to vegetables, fruits, meats, dairies, and fishes (von Braun 2007). Higher incomes, urbanization, and changing preferences are raising consumer domestic demands for high‐value products in developing countries. In Nigeria, the level of vegetable consumption is rising annually owing to greater appreciation of their food values (Haruna 2003). Furthermore, the availability of RTE vegetables in Nigerian markets is not equal and varies from one location to another and often depends on the local culture, socioe‐conomic conditions, food varieties, and dietary preferences. In addition, exotic vegetables such as Lettuce, Cabbages, and Cucumbers attract higher unit prices than traditional vegetables such as green *Amaranthus* and water leaf (Adeoye et al. 2010). ready‐to‐eat vegetables have posed a public health treat in Nigeria because different pathogenic microorganisms have been isolated from them (Bukar et al. 2010; Adeshina et al. 2012). The use of contaminated water from sewage sludge for the purpose of irrigation on agricultural soil has been found to increase the level of contamination of RTE vegetables (Oranusi and Olorunfemi 2011). In addition, the use of untreated water to wash vegetable produce, the continual sprinkling of vegetable produce (to keep them fresh in hot and dry seasons) with contaminated water, storage of RTE vegetables in contaminated places, and the use of contaminated containers from other farm produce to transfer RTE vegetables have been found to enhance their level of contamination (Singh et al. 2006; Mritunjay and Kumar 2015). The low incidence of *L. monocytogenes* contamination in the RTE vegetables across the traditional markets in South‐Western Nigeria reported in this work corroborates the European Food Safety Authority, (2014) baseline study in the European Union between 2010 and 2011, that though the pathogen can be isolated from a wide variety of foods, it is usually in relatively low numbers (<100/g). It also corroborates the report of Porto and Eiroa (2001) who recorded an incidence of 3.2% in RTE vegetables in Brazilian markets; Little et al. (2007), who found a 4.8% *L. monocytogenes* contamination in samples of raw vegetable salads in the United Kingdom; and Meloni et al. (2009) who found a 9.5% prevalence rate of *L. monocytogenes* in RTE foods collected from different outlets and processing plants in Sardina, Italy.

The differences in the level of contamination of the RTE vegetables in the states may be due to the different farming practices and postharvest handling methods adopted by farmers, the level of enlightenment by the retail sellers, exposure to environmental pollution, and storage temperatures of the RTE vegetables. Since there are no known standards for the preservation and selling ethics of RTE vegetables in Nigeria, most retail sellers of RTE vegetables in South‐Western Nigeria have street‐side stalls and only a few sell the vegetables in supermarkets. Street‐vended foods are perceived to be a major public health hazard in developing countries due to lack of basic infrastructures and facilities to maintain their diversity, mobility, and temporary nature which could initiate microbial contamination from sand, water dust, and air (Ekanem 1998; Rane 2011). A general lack of factual knowledge about the epidemiological significance of many street‐vended foods, poor knowledge of street vendors in basic food safety measures, and inadequate public awareness of hazards posed by certain RTE foods has severely fraught precise scientific approach to curb this serious issue of public health and safety (Rane 2011).

The higher incidence of *L. monocytogenes* in some markets such as Isikan, Bodija, Mushin, and Lafenwa may be due to the fact that the markets are urban. Sauders et al. (2012) investigated the diversity of different *Listeria* species in urban and natural environments in New York City and found that *L. monocytogenes* is more associated with urban environments. Furthermore, Baiyewu et al. (2007); Abbas and Jaber (2010) reported that urban markets are always wet and overcrowded and that higher incidences of *L. monocytogenes* may result from unhygienic handling, poor market practices, and diverse ecological niches of the pathogens. The higher incidence of *L. monocytogenes* in RTE vegetables in Lagos state in particular may be due to the fact that the state is overcrowded with a population estimate of about 21 million and a population density of more than 20,000 people/m^2^/land area thus resulting in high human activities such as dust, unclean water, improper handling, unhygienic market practices, and poor storage facilities that can contaminate the vegetables (Adedeji and Ademiluyi 2009). Similar result was obtained by Morobe et al. (2009) who found a prevalence of 4.3% *L. monocytogenes* in food samples in Gaborone South, Botswana and attributed it to overcrowding and ignorance on the part of the food handlers toward proper hygiene practices. Our investigations showed that cabbage harbored the highest incidence of *L. monocytogenes* while carrot had the least. Kovacevic et al. (2012) had earlier reported that the most contaminated group of RTE minimally processed vegetables were shredded cabbages which also contained the widest range of *Listeria* species. Ieren et al. (2013) also found that cabbage had the highest prevalence of *L. monocytogenes* in salad vegetables sold in Zaria, Nigeria. Furthermore, Monge and Arias‐ Echandi (1999) reported cabbage as a base vegetable for the occurrence of *L. monocytogenes*. Ieren et al. (2013) further attributed this to the presence of glucose, a fermentable sugar that can be readily utilized by the organism. The low incidence of *L. monocytogenes* in the carrots also agreed with the findings of Ieren et al. (2013) who found only 2.2% of *L. monocytogenes* isolates in carrots from five markets in Zaria metropolis. Zhu and Hussain (2014) also reported a low incidence of *Listeria spp* in carrots sold in Canterbury markets, New Zealand. Similar results were obtained by Sant'Ana et al. (2012) and they attributed the anti‐listeria activity of raw carrots to some antimicrobial compounds such as falcarinol, falcarindiol, and isocoumarin found in the carrot tissues. Beuchat and Brackett (1990) also found that the population of viable *L. monocytogenes* decreased upon contact with whole and shredded raw carrots and in cell suspensions in which raw carrots were dipped.

The higher incidence of *L. monocytogenes* in the RTE vegetables during the month of July corroborates previous reports that more *Listeria* spp. was isolated during the spring or low‐temperature seasons in the United Kingdom, Japan, and Turkey (MacGowan et al. 1994; Yoshida et al. 1999 and Atil et al. 2011). In South‐Western Nigeria, rain usually starts around March/April and continues till October. Owing to higher rainfalls during the month of July, most farmers prefer planting during this period. However, rain water carries a lot of human, environmental, animal wastes, and remains of decaying vegetation associated with high microbial risk (Obeng et al. 2007; Pavan da Silva et al. 2007) in which *L. monocytogenes* is usually a common habitat (Ikeh et al. 2010) as the organism is ubiquitous in outdoor environment (Hansen et al. 2006).

Although, the results obtained in this study indicated that the incidence of *L. monocytogenes* isolated from the RTE vegetables in traditional markets in South‐Western Nigeria was low, cabbage had the highest incidence of contamination while carrot had the least. Furthermore, there was a significant difference in the distribution of *L. monocytogenes* isolates in the month of July as compared with other months. It is also important to educate farmers on the need to cultivate good agricultural practices and also street hawkers of RTE vegetables on proper hygiene practices as the presence of this pathogen is a potential threat to public health.

## Conflict of Interest

The authors declare that they do not have any conflict of interest.
